# Emission Characteristics of NO_x_ and SO_2_ during the Combustion of Antibiotic Mycelial Residue

**DOI:** 10.3390/ijerph19031581

**Published:** 2022-01-29

**Authors:** Yaxin Ge, Guangyi Zhang, Jianling Zhang, Wennan Zhang, Lijie Cui

**Affiliations:** 1 School of Ecology and Environment, Beijing Technology and Business University, Beijing 100048, China; geyaxin14@mails.ucas.ac.cn; 2 School of Chemical Engineering, University of Chinese Academy of Sciences, Beijing 100049, China; 3 Institute of Process Engineering, Chinese Academy of Sciences, Beijing 100190, China; zhangjl@ipe.ac.cn; 4 Department of Chemistry and Molecular Biology, University of Gothenburg, 41296 Gothenburg, Sweden; 5 Department of Chemical Engineering, Mid Sweden University, 85170 Sundsvall, Sweden; wennan.zhang@miun.se

**Keywords:** antibiotic mycelial residue (AMR), combustion, NO_x_, SO_2_, CaO, desulfurization

## Abstract

The antibiotic mycelial residue (AMR) generated from cephalosporin C production is a hazardous organic waste, which is usually disposed of by landfilling that causes potential secondary environmental pollution. AMR combustion can be an effective method to treat AMR. In order to develop clean combustion technologies for safe disposal and energy recovery from various AMRs, the emission characteristics of NO_x_ and SO_2_ from AMR combustion were studied experimentally in this work. It was found that the fuel-N is constituted by 85% protein nitrogen and 15% inorganic nitrogen, and the fuel-S by 78% inorganic sulfur and 22% organic sulfur. Nitrogen oxide emissions mainly occur at the volatile combustion stage when the temperature rises to 400 °C, while the primary sulfur oxide emission appears at the char combustion stage above 400 °C. Increasing the combustion temperature and airflow cause higher NO_x_ emissions. High moisture content in AMR can significantly reduce the NO_x_ emission by lowering the combustion temperature and generating more reducing gases such as CO. For the SO_2_ emission, the combustion temperature (700 to 900 °C), airflow and AMR water content do not seem to exhibit obvious effects. The presence of CaO significantly inhibits SO_2_ emission, especially for the SO_2_ produced during the AMR char combustion because of the good control effect on the direct emission of inorganic SO_2_. Employing air/fuel staging technologies in combination with in-situ desulfurization by calcium oxide/salts added in the combustor with operation temperatures lower than 900 °C should be a potential technology for the clean disposal of AMRs.

## 1. Introduction

Antibiotic mycelial residues (AMRs) are the wastes generated from fermentative processes in antibiotics production [1]. Around one million tons of AMRs are generated each year in China—the major antibiotic producer in the world [2]. AMRs mainly consist of mycelia, surplus fermentative substrates, flocculant agents, intermediate metabolites, and residual antibiotics. They were frequently used as animal feed and plant fertilizer due to their high nutrition content [3]. However, AMRs have been listed in the National Catalog of Hazardous Wastes in China since 2008 [4], and various AMRs are forbidden as animal feed and plant fertilizer because residual antibiotics can easily accumulate in animals’ bodies, leading to the emergence of drug resistance [3,5,6].

So far, most AMRs are disposed of by landfilling, which causes potential secondary environmental pollution [7]. Other feasible and mature methods to treat AMRs have not been found; thus, antibiotic production is facing a big challenge. For the safe disposal of AMRs, many technologies are under research and development, including anaerobic digestion [8], hydrothermal treatment [3], microwave treatment [1], pyrolysis [2,9,10], gasification [11,12], combustion [13], and so on. Anaerobic digestion of AMRs is difficult since the residual antibiotics can suppress the microbiological process and even facilitate the development of bacterial antibiotic resistance [14,15]. Hydrothermal treatment (in combination with anaerobic digestion) and microwave treatment can effectively degrade hazardous antibiotics and lower the water content in AMRs [1,3]; however, they can not completely remove the organic materials in AMRs and bring out new challenges for disposing of the remaining solids with other environmental risks. In pyrolysis, solid materials remain to be treated further. In gasification, unmanageable viscous tar is generated. Generally speaking, the above-mentioned technologies are not mature, and the related reaction mechanisms and optimal reaction conditions are not clear [2,11,13].

For the disposal of solid waste from the pharmaceutical industry, combustion is the simplest and most commonly used method around the world. By combustion, solid wastes are safely disposed of, and the energy of the organic materials can be recovered simultaneously [16]. At present, a few antibiotic factories in China have started to employ combustion technology for AMR disposal [13]. AMRs have high N and S contents, which give rise to pollutant emissions from combustion. Many research works in the literature have investigated the N- and S-related emissions. Ma et al. [17,18] found that the combination of hydrothermal pretreatment and air-stage combustion can significantly reduce NO emissions. Zhang et al. [16] simulated the combustion of water-rich AMRs by adding steam to a fluidized bed and showed a clear decrease in NO emission by water vapor addition. On the other hand, SO_2_ emissions would be kept at a high concentration and might not be affected by steam. Ge et al. [19] also found that water in AMRs can decrease NO_x_ emissions during combustion in a fluidized bed. Jiang et al. [4] observed that the addition of AMRs into municipal waste did not lead to apparent increases in pollutant emissions. Wang et al. [20] investigated ash properties of the co-combustion of AMR with biomass. Gao et al. [21] simulated the co-combustion of AMR and biogas in a grate furnace. So far, many issues related to N and S emission characteristics remain unclear: distributions of N and S species in AMR, correlations between NO_x_/SO_2_ emissions with N/S species, detailed effects of combustion parameters, effectiveness of CaO desulfurization during combustion in a fixed bed, as well as effects of moisture in raw AMRs on the NO_x_ and SO_2_ emissions in comparison to the external steam addition. Thus, further works need to be done to improve combustion technologies for safe disposal and energy recovery from AMRs.

In this study, the N and S species in AMRs were characterized by X-ray photoelectron spectroscopy (XPS), and nitrogen and sulfur oxide emission characteristics during the AMR combustion were investigated by a TGA-MS setup and a fixed bed reactor with respect to the correlations between N/S species and NO_x_/SO_2_ emissions. Moreover, the effects of temperature, excess air, CaO addition, and water content of the fuels on the NO_x_ and SO_2_ emissions were studied in the fixed bed reactor. The mechanism of NO_x_/SO_2_ emissions during AMR combustion is clarified, and the effects of NO_x_/SO_2_ control methods are evaluated in this work, which provides valuable reference data for the industrial application of AMR combustion.

## 2. Materials and Methods

### 2.1. Feedstock

The AMR used in this work was supplied from a cephalosporin C production factory in Hebei Province, China. The moisture content in the raw AMR was about 70 wt.%. The ARM sample was firstly dried at 105 °C for 24 h, then pulverized to small particles, and finally sieved by a 0.2 mm diameter sieve. The results of the ultimate and proximate analyses of the dried AMR are shown in Table 1.

To characterize the composition of the AMR ash, the AMR was burned in an oven at 815 °C for 40 min (consistent with the method of the Chinese national standard of proximate analysis). The remaining ash was measured by an X-ray Fluorescence Spectrometer (XRF), and the results are shown in Table 2.

### 2.2. TGA-MS and XPS Instruments

A Thermalgravimetric Analyzer (TGA, LABSYS Evo, Setaram, Lyon, France) was connected to a Mass Spectrometer (MS, Tilon GRP Technology Limited, London, UK) [22] in order to simultaneously measure the mass loss and emitted gases (NO, NO_2_, and SO_2_) during pyrolysis or combustion of the AMR with increasing temperature. For each test, 10 ± 0.1 mg of the AMR sample was put into an alumina crucible and was subject to a hot gas flow at 100 mL/min with 100% Ar for pyrolysis or with 79% Ar and 21% O_2_ for combustion. The sample was heated from room temperature to 800 °C at a heating rate of 20 °C/min; the flue gases were detected and relatively quantified by MS.

With respect to the nitrogen and sulfur elements existing in the AMR, an XPS analyzer (ESCALAB250Xi, Thermo Fisher Scientific, Waltham, MA, USA) was used to characterize the related functional groups [18]. For XPS analysis, a sample was, in succession, dried in a vacuum dryer, degassed at 10^−7^ Pa for 3 h, and neutralized by Ar-ion sputtering. The sample was excited by Monochromatic Al K Alpha (350 W, hv = 1486.8 eV) X-ray. All spectra were obtained at 20 eV of pass energy in the fixed transmission mode. The resolution and analysis areas were 0.05 eV and 0.8 mm^2^, respectively. The analysis was automatically performed at 3–5 different positions on the surface to obtain high-quality N 1s and S 2p spectra.

### 2.3. Fixed Bed Combustor

**Figure 1:**
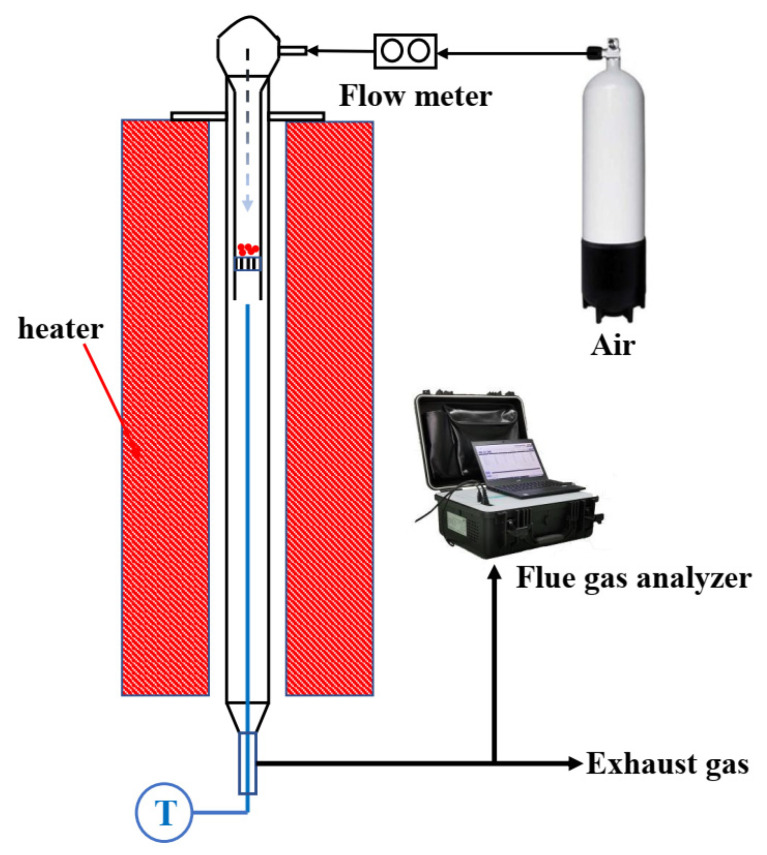
Schematic diagram of the fixed bed combustor.

The combustion tests were conducted in a fixed bed, which can be heated by an external electric heater, as shown in Figure 1. It consists of a fixed bed, a gas supply system, and a flue gas analyzer. The fixed bed consists of two parts, an inner reactor, and an outer reactor. The outer reactor is a quartz tube with a length of 70 cm, which provides enough long residence time for volatiles to be burned out. A thermocouple is inserted into the outer reactor from the bottom to measure the combustion temperature. The gas outlet is located at the bottom. The inner reactor is a shorter quartz tube with a length of around 25 cm; it has a sintered quartz porous plate at the bottom and a lid with a gas inlet on the top. The gas supply system consists of an air cylinder and a mass flow controller. A flue gas analyzer (Model 3080, Beijing SDL Technology, Beijing, China) equipped with a NO_x_ converter (convert NO_2_ to NO) is connected to the outlet of the outer reactor to monitor the online concentration of O_2_, CO_2_, NO_x_, and SO_2_ [16,17,18,19].

For the combustion tests in the fixed bed, 0.25 g AMR was blended with 5 g silica sand to maintain a certain bed height to enhance gas-solid interaction and prevent possible ash agglomeration at the plate during combustion [17]. The well-mixed sample was filled into the inner reactor and evenly tiled on the sintered quartz porous plate. Afterward, the top lid of the inner reactor was closed, and the gas flow was adjusted to the desired values of 1.0, 1.5, 2.0, or 3.0 L/min. Meanwhile, the outer reactor was heated up to the combustion temperature of 700 °C, 800 °C, or 900 °C, and the flue gas analyzer was ready to monitor the gas compositions. Finally, the inner reactor was rapidly inserted into the preheated outer reactor to initiate the AMR combustion. The combustion was completed until no CO/CO_2_ was released from the sample. Each experimental condition was repeated at least three times to ensure reliability. For CaO additive tests, 0.25 g AMR was blended evenly with 0.025–0.050 g CaO (that was, the Ca/S ratio of 2–4) and then mixed with 5 g silica sand. For the water additive tests, a desired amount of water was sprayed evenly by a small injector on the bed surface before the inner reactor was inserted into the outer reactor to make the water content in the sample reach a certain level of 9.1, 18.2, or 27.3%, respectively.

### 2.4. Gas Emission Calculation

For NO_x_, SO_2_, and CO, the gas emission rates were calculated by the following equation:

(1) $${ER}_{i}=\frac{C_{i}}{10^{6}}\;\times \;\frac{F}{60\times 22.4}{\;\times \;1000M}_{i}$$

where i denotes NO_x_, SO_2_, or CO, *ER*_i_ (mg/s) represents the gas emission rates, *C*_i_ (ppm) denotes the instantaneous concentration of each gas given by the flue gas analyzer, *F* is the total gas flow of 1.0, 1.5, 2.0, or 3.0 L/min, and *M*_i_ denotes the molar mass of the corresponding gas (NO_x_ uses the molar mass of NO).

The O_2_ consumption rate, reflecting the intensity of combustion, was calculated according to Equation (2):

(2) $$CR=\frac{{21\;-\;C}_{O}}{100}\;\times \;\frac{F\;}{60\;\times \;22.4}{\;\times \;1000M}_{O}$$

where *CR* (mg/s) represents the O_2_ consumption rates, *C*_O_ (%) denotes the instantaneous concentration of O_2_, *F* is the total flow of gas, and *M*_O_ indicates the molar mass of O_2_.

The total emission, *EA*_i_ *(mg)*, of NO_x_, SO_2_, or CO was calculated by Equation (3):

(3) $${EA}_{i\;}=\int_{0}^{T}{ER}_{i}(t)\;dt$$

where T and *t* represent the combustion duration and the instantaneous time, respectively.

The conversion ratio, *X*_i_, of N, or S, and the yield of CO, *X*_C_ were calculated by the following equation:

(4) $$X_{i}=\frac{{EA}_{i\;}{\times \;c}_{i}}{{\mathrm{Mass}\;\times \;f}_{i}}$$

where *c*_i_ (%) is the mass content of N in NO (S in SO_2_, C in CO). Mass denotes the mass of the added sample, 250 mg, *f*_i_ (%) is the elemental content (N, S, and C, as shown in Table 1) in the AMR sample.

The ratio of carbon not converted to CO, *B*_C_, which represents the burnout ratio of the fuel, was calculated by Equation (5):

(5) $$B_{C}=1\;-\;X_{C}$$

## 3. Results and Discussion

### 3.1. XPS and TGA-MS Analysis of AMR

**Figure 2:**
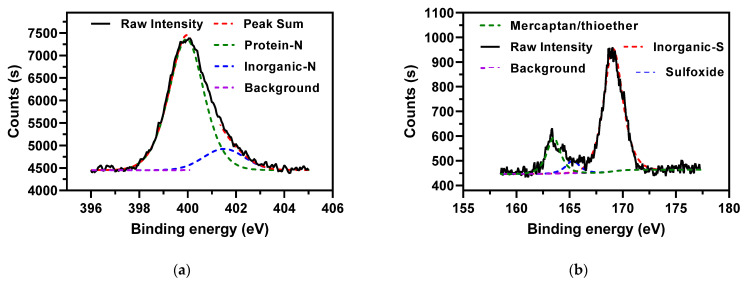
XPS Spectra for (**a**) N 1s and (**b**) S 2p in the AMR.

The N distributions over their species in the AMR feedstock are shown in Figure 2a and Table 3. The N-containing species in the AMR were dominated by protein nitrogen (399.9 eV) [23,24,25]. Such a high protein nitrogen content was attributed to a large proportion of abandoned fermentative substrates coming from high-protein plants, such as wheat and corn, in antibiotic production [13]. The minor N-containing species were characterized by inorganic nitrogen (401.5 eV) [23,24,25], mostly appearing in the form of NH_4_^+^-N [24].

For the S-containing species, the dominant species is inorganic sulfur (169.1 eV, 77.4%) [26], as shown in Figure 2b and Table 3. The other minor S-containing species include mercaptan/thioether (163.5 eV) and sulfoxide (165.3 eV) [26], which account for 16.8% and 5.8% of total sulfur, respectively. The two minor S-containing species were expected to be organic sulfur. However, herbaceous biomass usually has similar contents of organic and inorganic sulfur [27]. The high inorganic sulfur content surely originates from the addition of S-containing salts in the production of antibiotics.

**Figure 3:**
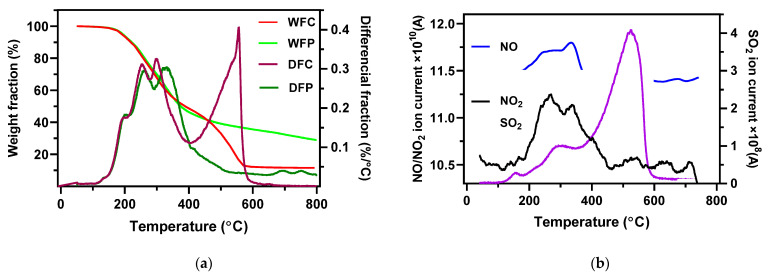
(**a**) Weight fraction and differential weight fraction as a function of temperature during pyrolysis and combustion of the AMR (WFC—weight fraction during combustion, WFP—weight fraction during pyrolysis, DFC–differential weight fraction during combustion, and DFP—differential weight fraction during pyrolysis). (**b**) The emission intensity of NO, NO_2_, and SO_2_ during AMR combustion in Ar/O_2_ atmosphere.

The mass loss of the AMR was registered by TGA against temperature increase, as shown in Figure 3a. The weight loss curves for the pyrolysis and combustion were similar below 400 °C, but highly different above 400 °C. Under the low temperature, AMR started to release volatile matter and was characterized by a typical biomass devolatilization stage for both pyrolysis and combustion [28]. In the case of combustion, char combustion and the decompositions of some inorganic materials in the AMR took place after the devolatilization stage and lost weight further with the second peak seen in the DFC curve.

The NO/NO_2_ emission trends against temperature present peak emission intensity between 200 and 400 °C, and leveled off above 400 °C, as shown in Figure 3b. These emission trends overlapped with the devolatilization stage, implying that the N-containing species were mainly contained in the volatiles of the AMR. The N-containing species of the AMR, i.e., protein nitrogen and inorganic nitrogen, given in Table 3, were easy to decompose at 200–400 °C [29,30]. Extremely low NO/NO_2_ emissions at the combustion temperatures above 400 °C suggested that the N-containing groups were not involved in the char formation (mainly by polymerization and cyclization reaction).

Different from NO/NO_2_ emissions, the SO_2_ emission trend shows two peaks. The first peak was small at about 300 °C and should be caused by the devolatilization of the AMR; thus, it can be attributed to organic sulfur [27,31]. The main SO_2_ emission occurred at the char combustion stage, which should be closely linked to the high content of the inorganic sulfur in the AMR [27,31].

### 3.2. Effect of Combustion Temperature

**Figure 4:**
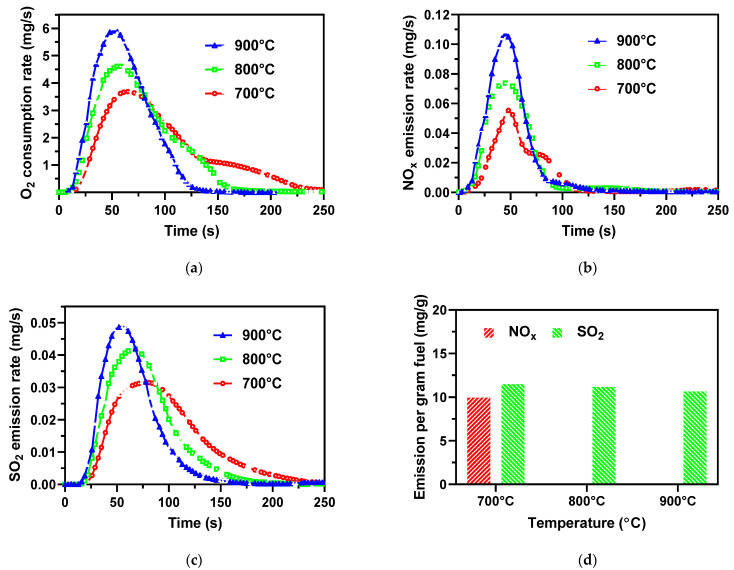
(**a**) O_2_ consumption, (**b**) NO_x_ emission, (**c**) SO_2_ emission as a function of time during combustion of the AMR, and (**d**) NO_x_ and SO_2_ emissions per gram AMR at different combustion temperatures.

Figure 4a shows the consumption of O_2_ against the combustion time, which can monitor the combustion process well. The real-time O_2_ consumption curve displays a unimodal distribution since combustions of both volatile and char take place continuously without staging as weight loss and simultaneously reach maximum combustion rate. The O_2_ consumption peak was higher at a higher combustion temperature and shifted to the left for a shorter combustion time, as seen in Figure 4a and Table 4. High temperatures also improve the burnout ratio of the AMR fuel, as given in Table 5.

As shown in Figure 4b,d, the NO_x_ emission curve also presents a unimodal distribution, and the peak and total amount of the NO_x_ emissions increased with increasing combustion temperature from 700 to 900 °C. High temperature was in favor of the fuel-N conversion to NO_x_ [31]. Comparing the O_2_ consumption curve, the time of the NO_x_ emission peak, *t*_max_, was short and did not have an obvious shift with increasing combustion temperature as shown in Table 4 and Figure 4a,b, which reasonably responds to the AMR fuel devolatilization stage, while O_2_ was consumed throughout the whole process, including both the volatile combustion and the char combustion. A similar result was observed by Zhao et al. from the algae biomass combustion experiment [31]. As shown in Figure 4d and Table 5, the conversion ratio of fuel-N to NO_x_, *X_N_*, increases from 6.9% to 11.9% as the temperature increases from 700 to 900 °C.

For the SO_2_ emission, Figure 4c shows similar curves to the O_2_ consumption curves in Figure 4a, suggesting a close relationship between the O_2_ consumption and the SO_2_ emission, much differently from NO_x_ emission. The oxidation of inorganic and organic sulfur overlapped in the rapid heating of the AMR samples in the fixed bed.

Slightly different from the O_2_ consumption, the SO_2_ emissions started later than the O_2_ consumption, and *t*_max_ of the SO_2_ emissions was longer than the O_2_ consumption, as also given in Table 4. Most of the sulfur (78%) in the AMR inorganic matter was decomposed and released significantly during char combustion at relatively high temperatures.

The higher combustion temperature causes faster and earlier SO_2_ emission and results in a shorter *t*_max_. On the other hand, the low temperature caused a slow heating rate and slow char combustion. Nevertheless, the total SO_2_ emission and the conversion ratio of fuel-S to SO_2_ (*X*_S_) were unchanged in spite of their different combustion temperatures, as presented in Figure 4d and Table 5. The releasable sulfur was sufficiently released at 600 °C, as indicated in the TGA-MS curve in Figure 3b. Due to the high content of calcium in the AMR ash seen in Table 2, sulfur should be tightly bonded with calcium to make it hard to be released at the typical combustion temperatures of 700–900 °C [32].

### 3.3. Effect of Excess Air

**Figure 5:**
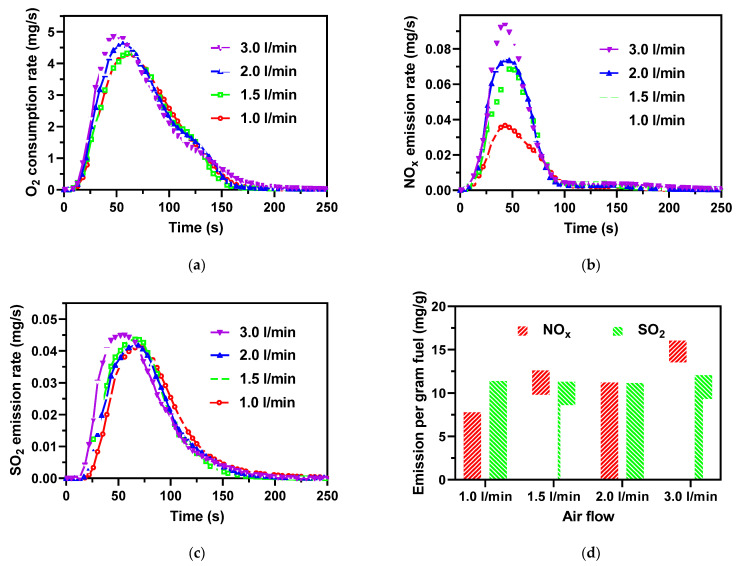
(**a**) O_2_ consumption, (**b**) NO_x_ emission, (**c**) SO_2_ emission as a function of time during combustion of the AMR, and (**d**) NO_x_ and SO_2_ emissions per gram AMR at different airflows.

The effect of airflow on the AMR combustion process is presented in Figure 5 with respect to O_2_ consumption, NO_x_, and SO_2_ emissions. The O_2_ consumption was not sensitive to airflow, but the peak shifted slightly to the left since higher excess air promotes volatile and char combustion. It should be mentioned that CO appeared at the airflow of 3 L/min due to the short residence time of the flue gas in the reactor, as given in Table 5.

For the NO_x_ emissions shown in Figure 5b, the peak appeared earlier than the O_2_ consumption in response to the AMR fuel devolatilization stage. The high airflow enhanced the oxidation of fuel-N to NO_x_ and, at the same time, inhibited the reduction of NO_x_ to N_2_. As a result, *X*_N_ increases from 5.4% at 1 L/min to 11.2% at 3 L/min, as shown in Figure 5d and Table 5. A lower air flow created an environment of O_2_ shortage, which could significantly reduce the formation of NO_x_. This suggests an effective method to control the NO_x_ emission from the AMR combustion via air staging combustion.

The SO_2_ emission had similar trends to the O_2_ consumption, as seen in Figure 5c. With increasing airflow, the SO_2_ emission peak slightly shifted to an earlier time, which might be related to the enhancement of the char combustion by excess air as the SO_2_ was mainly released during the char combustion. The SO_2_ emission peak lagged behind the O_2_ consumption peak, suggesting that the char combustion and ash decomposition dominated the SO_2_ emission. However, the airflow hardly changed the SO_2_ total emission, as shown in Figure 5d, and *X*_N_ was steadily kept at 23.8–25.1%, as listed in Table 5.

### 3.4. Desulfurization and Catalytic Effect of CaO

Most biomass fuels have a sulfur content below 0.2%, with only a few exceptions as high as 0.5–0.7% [33,34]. The S content of the AMR was extremely high, up to 2.9%, as seen in Table 1. Besides, the AMR seems to have a higher *X*_s_ (23.5% ± 1.5%) compared to other biomasses (5–20%) under similar combustion conditions [35]. Therefore, the addition of sorbent for desulphurization, like CaO into the AMR, is indispensable to lower the SO_2_ emission for AMR combustion.

**Figure 6:**
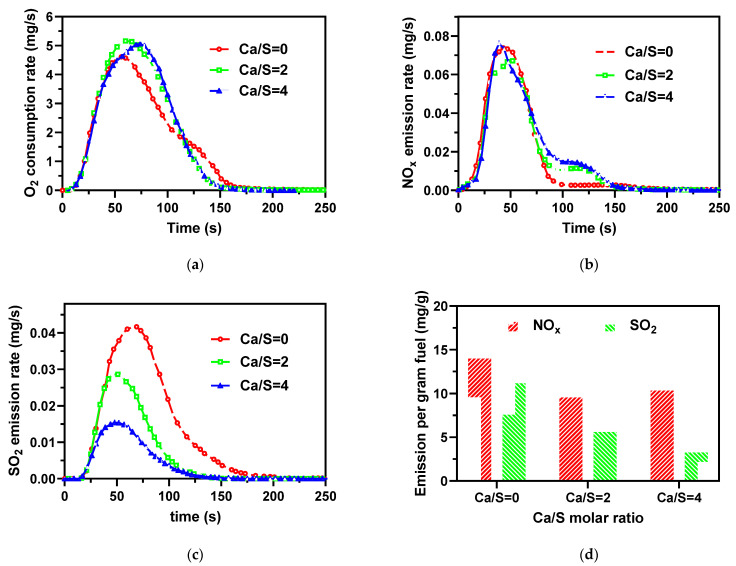
(**a**) O_2_ consumption, (**b**) NO_x_ emission, (**c**) SO_2_ emission as a function of time during combustion of the AMR, and (**d**) NO_x_ and SO_2_ emissions per gram AMR at different molar Ca/S ratios.

For the O_2_ consumption shown in Figure 6a, the curves nearly overlapped in the earlier combustion stage of 40 s. More CaO addition resulted in a greater O_2_ consumption in the later combustion stage of 50 to 110 s. CaO addition in the AMR also shortened the combustion time. The addition of CaO showed a positive effect on AMR combustion, especially on char combustion. This result agrees well with Yuan et al. [28], who concluded that calcium-based catalysis greatly affects char combustion but has minor effects on devolatilization.

NO_x_ emission and *t*_max_ were similar for the different Ca/S molar ratios, as presented in Table 4 and Figure 6b,d. Comparing SO_2_ emissions in Figure 6c, the NO_x_ started to be released at the earlier time of combustion and continued at a low level of NO_x_ emission during the combustion time of 100 to 150 s, which increased with the Ca/S molar ratio, as seen in Figure 6b. This may be due to the calcium-based compounds that increased the amount of char N during devolatilization [35,36]. Furthermore, the total amount of NO_x_ emission and the *X*_N_ did not change visibly, as shown in Figure 6d and Table 5.

CaO plays a significant role in sulfur retention, as shown in Figure 6c,d. The SO_2_ emission started at the same time but finished earlier at a higher Ca/S molar ratio. Moreover, a higher Ca/S molar ratio resulted in an earlier *t*_max_, as shown in Table 4. For the molar ratio of Ca/S = 2 and 4, the *t*_max_ of SO_2_ emissions were even shorter than the *t*_max_ of O_2_ consumption and approximately similar to the *t*_max_ of NO_x_ emission (40–50 s). In other words, the organic-S in the volatiles gradually dominated the SO_2_ emission with the increase of Ca/S. CaO additives can effectively control the SO_2_ emissions from inorganic-S. On the other hand, organic-S is released with the volatiles of the sample rather than directly released in SO_2_ form. Thus, sulfur at the devolatilization stage is hard to capture because of the poor gas-solid interaction in a fixed bed. The addition of CaO is apt to convert the inorganic sulfur into thermally stable CaSO_4_ and CaS [27,32,35]. Consequently, *X*_S_ decreased from 23.3 to 6.8% as the Ca/S molar ratio increased from 0 to 4, as listed in Table 5. In comparison, the desulfurization of the fixed bed was significantly worse than a fluidized bed [19] because the latter has a better gas-solid contact.

### 3.5. Effect of Water Content

**Figure 7:**
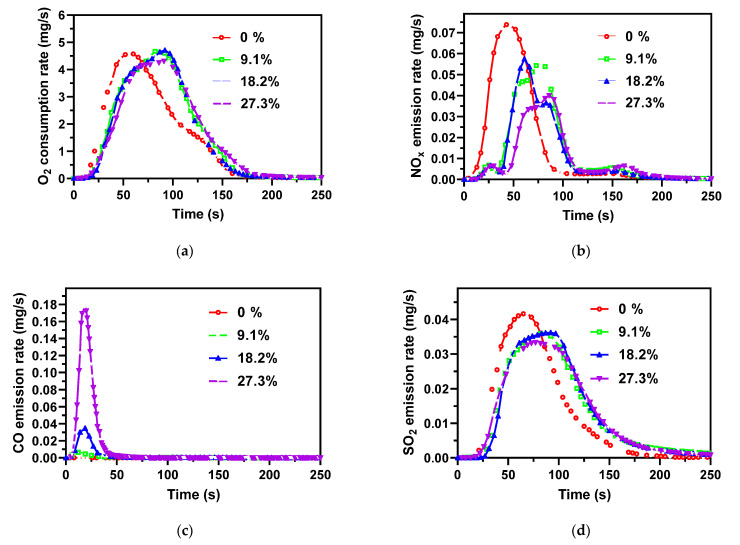

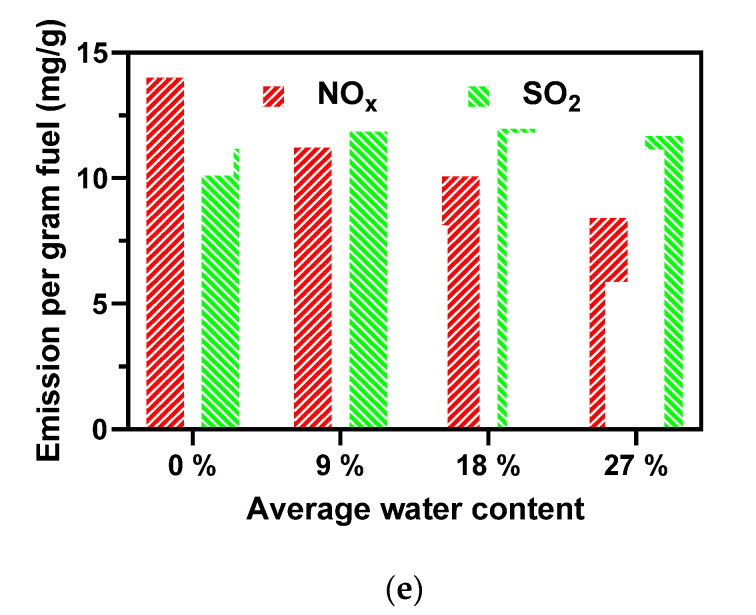
(**a**) O_2_ consumption, (**b**) NO_x_ emission, (**c**) CO emission, (**d**) SO_2_ emission as a function of time during combustion of AMR, and (**e**) NO_x_ and SO_2_ emissions per gram AMR at different water contents.

As shown in Figure 7a, the water addition significantly slowed down the AMR combustion. Generally speaking, the combustion of water-rich biomass undergoes drying, devolatilization, volatile and char combustion with the rising temperature of the fuel [37]. Because the drying process consumes a lot of heat to evaporate the water, the temperature of the fuel with a higher water content rises slowly, and the combustion duration has to be prolonged. The water-containing AMR samples release some CO in the earlier combustion stage of 50 s, and more CO is released as the water content increases. The CO was released earlier than the O_2_ consumption, following the earlier stage of devolatilization. Two aspects were involved in the CO release: (1) water vaporization makes a lower reactor temperature, thus hindering the burnout of samples, and (2) the formed water vapor increases the gas flow in the reactor and causes a shorter volatile resident time, leading to incomplete combustion. As a result, the burnout ratio decreased from 99.8% to 97.9% when the water content increased from 9.1% to 27.3%, as listed in Table 5.

The NO_x_ emission exhibited a bimodal distribution for the water-containing samples, as seen in Figure 7b, differing from the unimodal distribution for the dry samples. For the water-containing AMR, the first peak was smaller and occurred at 0–50 s, nearly synchronizing with CO release. The second NO_x_ emission peak was much higher and should be related to the volatile decomposition stage of the AMR combustion. The second NO_x_ emission peak contained two sub-peaks, which should be associated with the unstable combustion process for the water-containing samples, as shown by the O_2_ consumption curves. The occurrence of the first weak peak at 0–50 s might be explained by the devolatilization of the N-containing species, which is kept at a low level by the high concentration of CO and thus the reduction effect of CO on NO_x_ (2NO + 2CO = N_2_ +2CO_2_) [16,37]. Thus, NO_x_ reaches a small peak (at 25 s), then decreases due to the reducing atmosphere, and then increases again at 40–45 s because the reducing matters are consumed by O_2_. In conclusion, higher water content reduces the conversion of the fuel-N to NO_x_, as shown in Figure 7e and Table 5.

As Figure 7d illustrates, the SO_2_ emission from combustion of the water-containing fuel sample had a similar unimodal distribution to that from the combustion of the dry AMR sample, but with a time delay due to evaporation of water in advance. The increase in water content did not seem to reduce the total amount of SO_2_ emissions from the AMR combustion in the present case of the fixed bed, as shown in Figure 7e and Table 5. Zhang et al. [16] reported that steam could promote the capture of SO_2_ by the AMR ash in a continuous feeding fluidized bed. In the case of a fixed bed, the water was evaporated to form steam and escaped from the sample, and the chance of steam reacting with sulfur-containing species during the char combustion process was very small. In addition, compared to a continuous feeding fluidized bed, a fixed bed did not have much ash to capture SO_2_.

## 4. Conclusions

The emission characteristics of NO_x_ and SO_2_ from AMR combustion were investigated based on an experimental study conducted in a fixed bed. Analysis of the AMR showed that the fuel-N was constituted by 85% protein nitrogen and 15% inorganic nitrogen and the fuel-S by 78% inorganic sulfur and 22% organic sulfur. The volatile combustion resulted in most NO_x_ when the temperature rose to 400 °C, while the primary sulfur oxide emission occurred at the char combustion stage above 400 °C. Increasing the combustion temperature and airflow caused higher NO_x_ emissions. The addition of CaO changed the distribution of NO_x_ emission by converting part of the volatile nitrogen into char nitrogen. High moisture content in AMR significantly reduced the NO_x_ emission by lowering the combustion temperature and generating more reducing gases such as CO. For the SO_2_ emission, the combustion temperature (700 to 900 °C), airflow, and AMR water content did not seem to exhibit obvious effects. The presence of CaO significantly inhibited SO_2_ emission, especially for the SO_2_ produced during the AMR char combustion. In addition, low combustion temperatures, high water contents in the fuel, as well as low airflows were unfavorable for the burnout of the AMR. Therefore, the direct combustion of AMR with in-situ NO_x_ control must be balanced with fuel burnout. Employing air/fuel staging technologies in combination with in situ desulfurization by calcium oxide/salts, in addition to operating temperatures lower than 900 °C, could be a potential technology for the clean disposal of AMRs. In addition, a number of aspects will require further attention and dedicated experimental work. In particular, systematic studies of air/fuel staging technologies and continuous feed tests need to be carried out.

## Figures and Tables

**Table 1:** Fuel properties of the dried AMR material.

**Proximate Analysis (wt.%, as Received)**	
Moisture	2.8
Volatile matter	85.0
Ash	10.2
Fixed carbon	2.0
**Ultimate analysis (wt.%, dry ash-free)**	
C	57.7
H	9.1
N	7.9
O	22.3
S	2.9
High Heating Value (MJ/kg)	23.58

**Table 2:** Composition of the AMR ash.

Species	SO_3_	CaO	P_2_O_5_	K_2_O	SiO_2_	MgO	Na_2_O	Al_2_O_3_	Fe_2_O_3_	SrO	Total
Content (wt.%)	40.83	35.02	14.62	3.74	2.27	1.12	0.90	0.65	0.40	0.002	99.74

**Table 3:** N and S distributions over different N and S species in the AMR sample.

Elements	Element Species	Content (%)
N	Protein nitrogen	84.6
	Inorganic nitrogen	15.4
S	Mercaptan/thioether	16.8
	Sulfoxide	5.8
	Inorganic sulfur	77.4

**Table 4:** The time of curve peak, *t*_max_, under various combustion conditions for O_2_ consumption, NO_x_ emission, and SO_2_ emission.

Gas Curve	O_2_ Consumption	NO_x_ Emission	SO_2_ Emission
*t*_max_ (s) (700 °C, 2.0 L/min, Ca/S = 0, dry)	65	49	81
*t*_max_ (s) (800 °C, 2.0 L/min, Ca/S = 0, dry)	56	44	68
*t*_max_ (s) (900 °C, 2.0 L/min, Ca/S = 0, dry)	52	46	58
*t*_max_ (s) (800 °C, 1.0 L/min, Ca/S = 0, dry)	61	43	71
*t*_max_ (s) (800 °C, 1.5 L/min, Ca/S = 0, dry)	61	48	68
*t*_max_ (s) (800 °C, 3.0 L/min, Ca/S = 0, dry)	51	42	52
*t*_max_ (s) (800 °C, 2.0 L/min, Ca/S = 2, dry)	61	50	46
*t*_max_ (s) (800 °C, 2.0 L/min, Ca/S = 4, dry)	73	40	48

**Table 5:** Conversion ratio of fuel-N (fuel-S) to NO_x_ (SO_2_) and C burnout ratio under different experimental conditions.

Combustion Condition	Fuel-N to NO_x_, *X*_N_	Fuel-N to SO_2_, *X*_S_	Burn out Ratio, *X*_N_
700 °C, 2.0 L/min, Ca/S = 0, dry	6.9	23.9	98.8
800 °C, 2.0 L/min, Ca/S = 0, dry	9.7	23.3	100
900 °C, 2.0 L/min, Ca/S = 0, dry	11.9	22.2	100
800 °C, 1.0 L/min, Ca/S = 0, dry	5.4	23.8	100
800 °C, 1.5 L/min, Ca/S = 0, dry	8.8	23.6	100
800 °C, 3.0 L/min, Ca/S = 0, dry	11.2	25.1	99.9
800 °C, 2.0 L/min, Ca/S = 2, dry	9.7	11.7	100
800 °C, 2.0 L/min, Ca/S = 4, dry	10.4	6.8	100
800 °C, 2.0 L/min, Ca/S = 0, 9.1%	7.8	24.1	99.8
800 °C, 2.0 L/min, Ca/S = 0, 18.2%	7.0	24.9	99.5
800 °C, 2.0 L/min, Ca/S = 0, 27.3%	5.9	24.3	99.5

## Data Availability

Not applicable.
